# Bond Strength of Silane-Containing Universal Bonding Agents to Lithium Disilicate

**DOI:** 10.4317/jced.62011

**Published:** 2025-04-01

**Authors:** Daniel L. Tannen, Adam J. Wallum, Terrell M Mitchell, James J. Renda, Kraig S. Vandewalle

**Affiliations:** 1 DDS, MS. Capt, USAF, DC. General Dentist. Dental Clinic. 208 W. D.L. Ingram Avenue, Cannon AFB, NM 88103; 2 DMD, MS. Maj, USAF, DC. Director, Restorative Dentistry. Advanced Education in General Dentistry Residency. AF Postgraduate Dental School. 1615 Truemper St. Joint Base San Antonio - Lackland, TX, USA 78236. Uniformed Services University of the Health Sciences, Bethesda, MD, USA; 3 DDS, MS. Lt Col, USAF, DC. Training Officer. Advanced Education in General Dentistry Residency. AF Postgraduate Dental School. 1615 Truemper St. Joint Base San Antonio - Lackland, TX, USA 78236. Uniformed Services University of the Health Sciences, Bethesda, MD, USA; 4 DMD, MS. Col, USAF, DC. Program Director. Advanced Education in General Dentistry Residency. AF Postgraduate Dental School. 1615 Truemper St. Joint Base San Antonio - Lackland, TX, USA 78236. Uniformed Services University of the Health Sciences, Bethesda, MD, USA; 5 DDS, MS. Col (ret), USAF, DC. Air Force Consultant in Dental Research. Advanced Education in General Dentistry Residency. AF Postgraduate Dental School. 1615 Truemper St. Joint Base San Antonio - Lackland, TX, USA 78236. Uniformed Services University of the Health Sciences, Bethesda, MD, USA

## Abstract

**Background:**

The purpose of this study was to evaluate the microshear bond strength to lithium disilicate of four silane-containing universal adhesives (SUAs): Scotchbond Universal Adhesive (3M), Scotchbond Universal Adhesive Plus (3M), Clearfil Universal Bond Quick (Kuraray), and Universal Bond II (Tokuyama), with or without the use of a separate silane primer.

**Material and Methods:**

Lithium disilicate blocks (IPS e.max CAD, Ivoclar) were sectioned to create 4 equal partitions. Blocks were then crystallized and mounted in polyvinyl chloride (PVC) pipes. The blocks were steam cleaned, subjected to 5% hydrofluoric acid etch, rinsed, and prepared with or without silane application. Resin cement was applied to prepared blocks within standardized silicone tubing matrices and light-cured forming four resin cement specimens per block and twelve specimens per group (n=12). The dual-cure resin cement with and without the use of silane served as a positive and negative control, respectively. The tubing was removed and then the specimens were subjected to 500 thermocycles. The specimens were loaded perpendicularly in a universal testing machine with a shear force until bonding failure. The mean microshear bond strength was calculated per group and analyzed with ANOVA and Tukey’s post hoc tests (alpha=0.05). Following testing, each specimen was examined to determine failure mode.

**Results:**

Except for Universal Bond II, an application of silane resulted in a significant increase in bond strength (*p*<0.05) when using the SUAs. Without the use of a silane, Universal Bond II had significantly greater bond strength (*p*<0.0001) than all other SUAs. All the SUAs without the use of silane resulted in lower bond strength (except UB) compared to the typical clinical practice of the use of silane alone. Greater mixed and cohesive failures were observed with groups that used silane.

**Conclusions:**

Silane-containing universal bonding agents are being marketed in lieu of silane application on the intaglio surface of lithium disilicate prior to cementation. The results from this study indicate that using SUAs (except Universal Bond II) in this manner may decrease bond strength of the interface between lithium disilicate and resin cement.

** Key words:**Universal bonding agent, silane, lithium disilicate, bond strength.

## Introduction

The rehabilitation of dentition with non-metallic materials has increased in popularity with both patient and provider preference in recent years (1). For single-unit anterior prostheses, the top material of choice for surveyed dentists in the National Dental Practice-Based Research Network was lithium disilicate. For single-unit posterior prostheses, lithium disilicate was among the top 3 choices (2). The application of adequate surface treatment of these glassy-ceramics is one of the main factors influencing the adhesion performance and long-term clinical survival when bonded with resin-based materials (3,4). The historically accepted resin-ceramic surface treatment for glassy-ceramics includes hydrofluoric acid (HF) etch and rinse followed by a separate silane application (5).

Application of HF partly dissolves the glassy phase exposing the crystalline structure and creates a rough surface for micromechanical retention. The result is a prosthesis intaglio with high surface energy allowing for the efficient application of glass ceramic primers containing silane (6-8). Silane is a bifunctional molecule that promotes adhesion between resin cements and ceramics via covalent bonds. The silanol (Si-OH) group in silane creates a covalent bond to the exposed Si-OH group on etched glass- ceramics creating strong siloxane (-Si-O-Si-) linkages. The terminal C=C functional group of silane forms new C-C sigma bonds with the methacrylate in resin via free-radical polymerization (9). As a result, resin composite and the substrate surface are connected by the silane coupling agent (10). To simplify the cementation protocol of glass-ceramic restorations, manufacturers have developed universal adhesive systems by combining primers with functional molecules (e.g., silane and 10- methacryloyloxydecyl dihydrogen phosphate [10-MDP] monomer) into a simplified system often utilizing a single bottle. These products advertise utilization in all restorative needs, including the surface treatment of glassy ceramics.

Although simplifying adhesive formulations by incorporating silane theoretically decreases steps or required products in clinical cementation protocols, there is evidence that separate silanization is required even if an SUA is used (11). A recent systematic review and meta-analysis of *in vitro* studies found that a silane-containing universal adhesive (Scotchbond Universal, 3M ESPE, St. Paul, MN) was not as effective as a glass-ceramic primer (silane) in promoting bond strength to glass ceramic (12). SUAs are more acidic than glass-ceramic primers. The acidic water-based solution of SUAs hydrolyze silanol groups (-Si-OH), and a self-condensation reaction may occur between the silanols of neighboring molecules, forming oligomers that can no longer bond to glass-ceramics (13,14). Recently, 3M has released Scotchbond Universal Adhesive Plus containing an organo-silane, 3-aminopropyltriethoxysilane (APTES) in addition to the original silane, γ- methacryloxypropyltriethoxysilane (γMPTES) in the original Scotchbond Universal. The company claims this addition improves glass-ceramic adhesion (15). Universal Bond II (Tokuyama, Tokyo, Japan) claims to avoid this problem by means of a two-bottle system (16). Kuraray (Tokyo, Japan) has recently updated their silane-containing universal bonding agent, Clearfil Universal Bond Quick to include a new amide monomer with claims of decreased technique sensitivity and application time (17). Regarding the newly introduced SUAs, is the addition the organo-silane in Scotchbond Universal Adhesive Plus superior to the existing formulation and has the development of this new monomer overcome the degradation of silanes seen in other SUAs? Does the two-bottle system employed by Tokuyama Universal Bond II improve glass-ceramic adhesion?

Overall, there are limited studies exploring the novel claims of these newer formulations of SUAs. The purpose of this study was to evaluate the microshear bond strength and failure mode of a resin cement to lithium disilicate of four SUAs with or without the use of a silane primer. The resin cement with silane primer served as positive control and is referred to as ‘typical clinical practice.’ The resin cement without any primer (silane or SUA) served as a negative control. To address manufacturer claims and evaluate laboratory performance, three null hypotheses were examined. The first null hypothesis: there would be no difference in microshear bond strength of a resin cement to lithium disilicate based on surface silane treatment (SUA alone vs SUA with separate silane application). The second null hypothesis: there would be no difference in microshear bond strength of a resin cement to lithium disilicate based on the material type used. The third null hypothesis: there would be no difference in microshear bond strength of a resin cement to lithium disilicate of an SUA compared to the control.

## Material and Methods

Ten groups of 12 specimens each were created and divided into silanated and unsilanated subgroups for a total of 120 specimens. Lithium disilicate blocks (IPS e.max CAD, Ivoclar, Schaan, Liechtenstein) were sectioned in half using a precision saw (IsoMet 5000, Buehler, Lake Bluff, IL) with a diamond-impregnated disk. Blocks were then partially sectioned into 4 equal partitions creating 4 specimens per block. Blocks were crystallized in a ceramic oven (Programat P500, Ivoclar) according to manufacturer’s instructions and mounted in 1-inch polyvinyl chloride pipes with clear acrylic (Vitacrilic, Fricke International, Streamwood, IL). Blocks were steam cleaned and subjected to 5% hydrofluoric acid etch (IPS Ceramic Etching Gel, Ivoclar) for 20 seconds and rinsed with water. The blocks were randomly allocated into the groups and prepared with and without a separate application of a silane-containing primer (Bis-Silane, Bisco, Schaumburg, IL).

An adhesive resin cement (NX3, Kerr Dental, Orange, CA) was bonded to lithium disilicate blocks using the following four SUAs: Scotchbond Universal (SBU), Scotchbond Universal Plus (SBU+), Universal Bond II (UB), and Clearfil Universal Bond Quick (CU) with and without silane. In addition, NX3 was bonded to the lithium disilicate block without an SUA - with and without silane - and served as a positive control and negative control groups, respectively. The application instructions and components for the materials are listed in  Table 1.

Silicone tubing was cut to produce 1-2 mm high matrices with internal diameters of 2 mm for standardization. The matrices were filled with an automixed dual-cure resin cement (NX3) and applied to the prepared lithium disilicate specimens. Matrices were held firmly in place and polymerized on all surfaces with a light curing unit (Valo Grand, Ultradent Products, South Jordan, UT) for 20 seconds each. The specimens were stored in a closed container in a humid environment using distilled water-soaked paper towels at room temperature for 48 hours. After 48 hours, the specimens were subjected to 500 cycles of thermocycling in distilled water at 5 and 55°C with a dwell time of 30 seconds at each temperature (Sabri Dental Enterprise, Downers Grove, IL).

**Figure 1:**
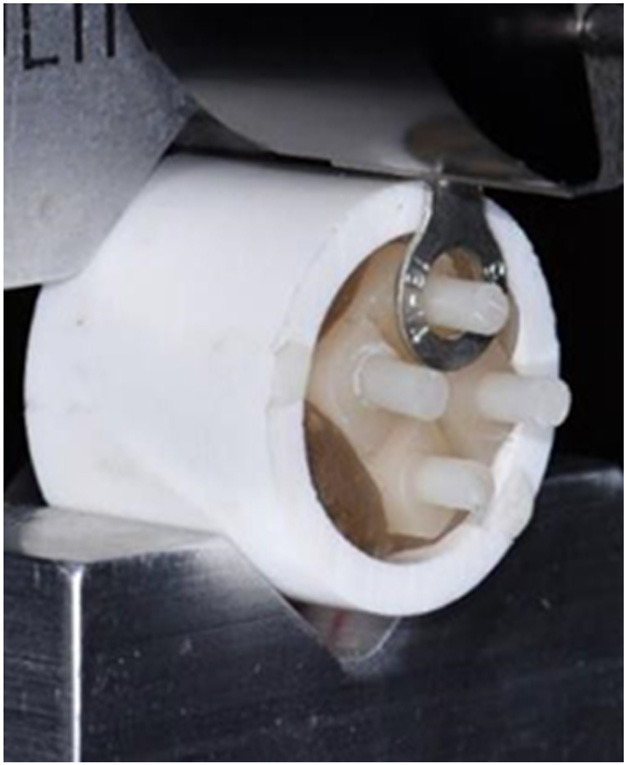
Block mounted in universal testing machine. Resin cement button loaded in shear at the resin cement/lithium disilicate interface.

To test microshear bond strength, specimens were loaded perpendicularly in a universal testing machine (Model 5943, Instron, Norwood, MA). A shear force at a crosshead speed of 1mm/min was applied with a 16-gauge ring (Utilitech, Cincinnati, OH) placed around each resin cement button at the resin cement/lithium disilicate interface as shown in Figure 1. Following testing, each specimen was examined using a 10x stereomicroscope (SMZ-1B, Nikon, Melville, NY, USA) to determine if the failure mode was an adhesive fracture at the resin cement – ceramic interface, a cohesive fracture in the resin cement, or a mixed (combined adhesive and cohesive) fracture.

Microshear bond strength values in megapascals (MPa) were calculated from the peak load of failure (Newtons) divided by the specimen surface area. The mean microshear bond strength and standard deviation was determined for each group. Data were analyzed using a two-way analysis of variance (ANOVA) and Tukey’s post hoc tests to evaluate the effect of a separate silane application (2-levels) or material (5-levels) on the microshear bond strength of the resin cement to lithium disilicate (alpha=0.05) using statistical software (SPSS version 25, IBM, Chicago, IL). The data were subsequently analyzed with multiple one-way ANOVAs and Tukey’s post hoc tests per surface silane treatment or unpaired t-test per material. In addition, the positive control group, NX3 resin cement with silane, was compared to the SUA bond strengths without silane using a Dunnett’s multiple comparison test (alpha = 0.05).

## Results

The results of the two-way ANOVA found significant differences in microshear bond strength based on separate silane application (*p*<0.001) and on material (*p*<0.001) with significant interactions (*p*<0.001). The microshear bond strengths of the resin cement to the lithium disilicate using the various SUAs are shown in  Table 2. An application of silane resulted in a significant increase in microshear bond strength (*p*<0.001) except when using UB. There was no significant difference (*p*=0.572) in microshear bond strength with (13.93 ± 3.89 MPa) or without (12.88 ± 5.01 MPa) the use of silane with UB. Without silane, UB resulted in the greatest microshear bond strength of the resin cement to the lithium disilicate (12.88 ± 5.01 MPa) and was significantly greater (*p*<0.001) than all other materials. The resin cement (NX3) when serving as a negative control, had the lowest microshear bond strength (0.63 ± 0.46 MPa), but it was not significantly different from the SUAs: CU (1.20 ± 0.53 MPa; *p*=1.00), SBU (1.48 ± 0.43 MPa; *p*=0.907) and SBU+ (1.68 ± 0.42 MPa; *p*=0.835) without silane. With silane, UB had the greatest microshear bond strength (13.93 ± 3.89 MPa), but it was not significantly different from SBU (14.00 ± 4.39 MPa; *p*=0.997) or CU (12.55 ± 4.33 MPa; *p*=0.892). NX3, when serving as a positive control (5.19 ± 3.24 MPa) and SBU+ (8.27 ± 2.30 MPa) were not significantly different (*p*=0.835) from each other; but both were significantly lower (*p*<0.003) than the other SUAs with the use of silane. The positive control, NX3 resin cement with silane, had significantly different microshear bond strength values compared to the SUAs without silane: greater than SBU, SBU+, and CU but significantly lower than UB (*p*<0.0003).

**Figure 2:**
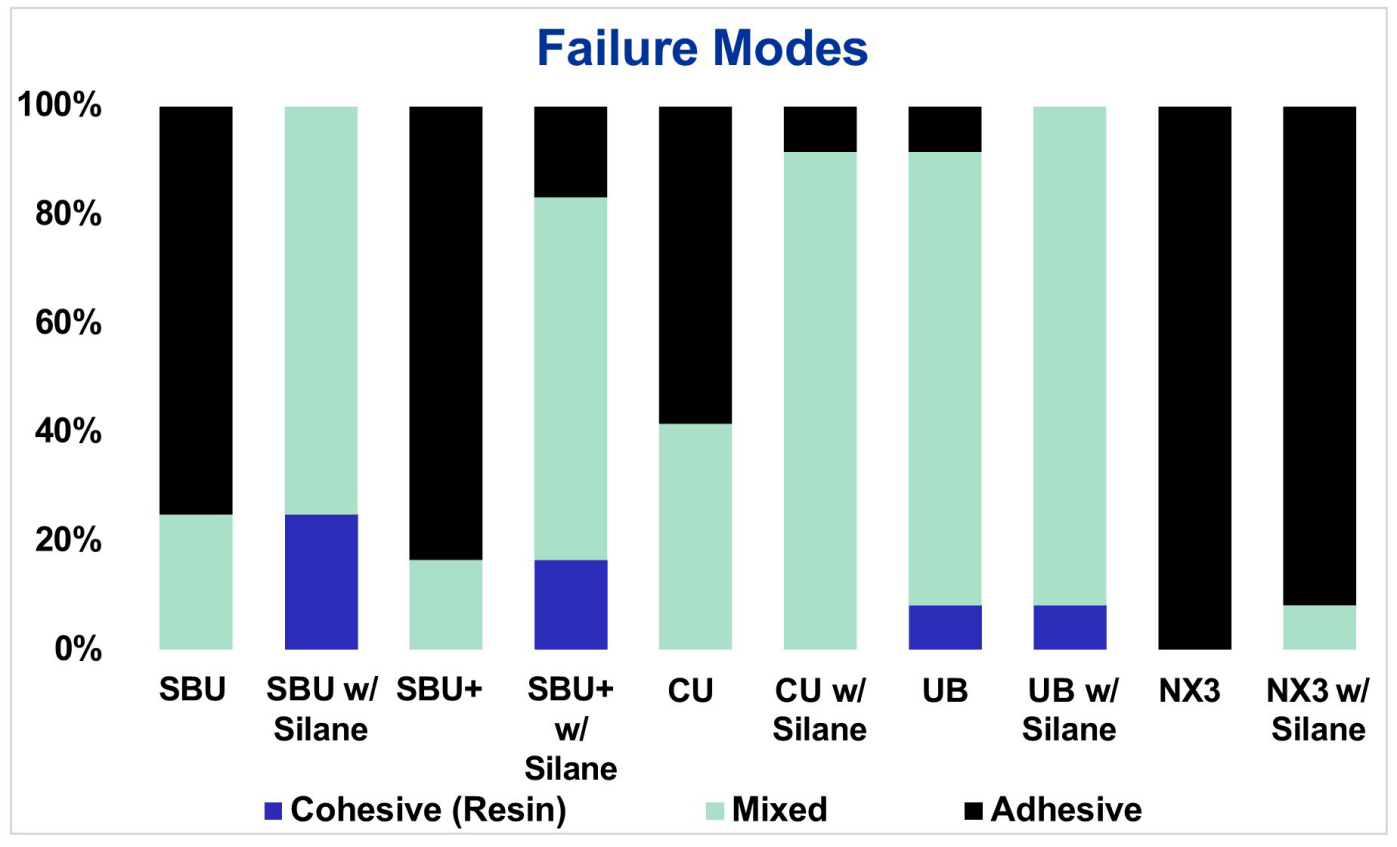
Failure modes of the various groups.

As shown in Figure 2, there were fewer adhesive fractures and more mixed or cohesive fractures in groups with a separate silane application.

## Discussion

The aim of this study was to evaluate the microshear bond strength of a resin cement to lithium disilicate utilizing four SUAs with and without a separate silane application.

The first null hypothesis was rejected: differences in microshear bond strength were found between groups based on the use of silane. Each SUA had statistically significant improvement in microshear bond strength with separate silane application, except UB. When used as instructed by the manufacturer, there was no difference in microshear bond strength when UB was used alone or in conjunction with a silane primer. Except UB, these results concur with conclusions of systematic reviews of laboratory studies which suggest a separate silane application when using Universal Adhesives (11,12). The two-bottle delivery system by Tokuyama Universal Bond maintains the acidic monomer (Bond A) and ceramic primers (Bond B) separate until just prior to application, potentially minimizing silane deterioration (16).

The second null hypothesis was also rejected: differences in microshear bond strength were found based on material type. Without silane, UB demonstrated a greater microshear bond strength. As discussed in the previous section, it is proposed that the greater microshear bond strength is likely due to the two-part delivery system. When used without silane, there was no difference in microshear bond strength between CU, SBU, and SBU+. With silane, SBU, CU, and UB demonstrated greater microshear bond strength compared to SBU+ and the positive control.

Interestingly, of the universal adhesives tested, SBU+ was the only true one-bottle system. Some differences in performance may be attributed to this simplification. UB is delivered as a two-bottle system and although SBU and CU are packaged as one-bottle systems, they both require respective dual-cure activators (DCAs) per manufacturer IFUs for this application. A study evaluating SBU found improved microshear bond strength when using a dual cure activator versus use of SBU alone (18). A possible explanation for this is due to the rapid hydrolytic process of silane when it reaches a pH that is lower than its range of highest stability: pH 4 to 5 (3,20,21). The pH of SBU is 2.7 – 3.0 to enable self-etching modalities. al-Salehi *et al*. suggests that the addition of a more alkaline dual-cure activator may increase the pH of the mixture and improve bonding compared to use of SBU without dual-cure activator (18). This may explain the improved performance of SBU and CU which require a dual-cure activator, compared to SBU+, which does not. It is important to note that minimizing silane degradation with a DCA is only applicable when using silane in addition to an SUA. When utilizing an SUA (besides UB) per manufacturer’s instructions, available silane has potentially already degraded due to the acidity required for self-etching modalities.

The third null hypothesis was rejected: differences in microshear bond strength were found between the use of an SUA compared to the positive control. A common intaglio preparation when bonding lithium disilicate includes HF etch, application of a silane primer and use of a resin cement (positive control). In this study, this is referred to as ‘typical practice’ or ‘standard of care.’ Compared to the standard of care, and when used as instructed, SBU, SBU+, and CU had lower microshear bond strength. When used as instructed, UB demonstrated greater microshear bond strength than the typical practice (*p*<0.05). When used with silane, the results of this study suggest the addition of a SUA (specifically UB, CU, and SBU) improved bond strength over the typical practice of silane plus resin cement. These findings present an interesting, although limited, perspective on potential improvement of bond strength for non-retentive restorations. More studies challenging this finding will be required especially due to the limitations associated with this study.

Regarding failure modes of tested groups: overall the results are consistent with expectations given the microshear bond strength values. Mixed or cohesive failure modes are often associated with higher bond strength compared to purely adhesive failure modes (19). When used without silane, there were more adhesive failures associated with all SUAs (Fig. 2). As anticipated, the application of silane used in conjunction with an SUA led to a decrease in the number of adhesive failures and an increase in mixed and cohesive failures.

There are some differences between the findings of this study and other laboratory studies and some systematic reviews concerning the long-term stability of the bond strength of universal adhesives. A recent study observed that the immediate bond strength of universal adhesives to feldspathic ceramics was improved by a separate silane coupling agent application; but found a significant decrease (25.5 – 40%) in bond strength after 6 months of water aging. In that study, the group of universal adhesives that were applied without silane-maintained bond strength after 6 months of aging. At 6 months there was no significant difference between groups that used silane, and those that did not (20). The goal of that study was to evaluate repair of feldspathic ceramic with a resin composite and utilized different SUAs than this study. Although direct comparisons cannot be made, the study raises important questions about bond strength retention of SUAs.

In a recent systematic review of laboratory studies, the authors concluded that the application of an adhesive layer did not improve the long-term bond strength of etched and silanized glass-ceramics (21). These conclusions are not coincident with the findings of this short-term study. Of the 14 studies included in the meta-analysis of the forementioned systematic review, only 2 included an SUA for more direct comparison: SBU. The two studies evaluated microshear bond strength of a resin-based material to lithium disilicate at 24 hours of water storage (immediate) versus 1-year (aged). Among other tested groups, one of the two studies compared the mean microshear bond strength of an adhesive resin cement to lithium disilicate with a non-MDP containing silane and with SBU with or without silane. The results of that study indicated that SBU alone and silane alone had greater mean bond strength compared to silane plus SBU after 24 hours but found no difference in microshear bond strength of these groups after 1 year of water storage (22). The other study compared the microshear bond strength of a flowable composite to lithium disilicate with silane and with SBU with or without silane. Among other tested groups, silane plus SBU demonstrated greater microshear bond strength than both SBU alone and silane alone at 24 hours. Silane plus SBU maintained greater microshear bond strength at 1 year than SBU alone but was less than the silane control (23).

A possible mechanism for the differences between the conclusions of the systematic review and this study may be due in part to the utilization of limited aging. Adhesives contain hydrophilic monomers and solvents that can increase water sorption at the interface. In groups that included adhesive application, that adhesive layer may be more susceptible to hydrolytic degradation and water sorption over time. In groups that did not include adhesive application, if the resin cement is able to adequately wet the silanized ceramic surface, maturation of the bond can occur even with long-term water immersion (24). This suggests that with more time, the bond in the NX3 plus silane group may continue to mature relative to degradation of the NX3 plus silane and SUA groups. With longer aging procedures, the differences observed in this study that suggest an adhesive layer improved the bond strength of etched and silanized glass-ceramics may become less significant over time. While other studies in the systematic review utilized 5,000 or more thermocycles, or stored specimens for up to a year, this study used 500 thermocycles (21). Piloting demonstrated a higher number of thermocycles would lead to pretesting specimen failure due to the lower bond strength observed in the groups where SUA was used alone ( Table 2). It is important to recognize that there were no studies included in the forementioned systematic review that included newer released SBUs such as SBU+, UB, and CU. This highlights a need for more studies challenging manufacture claims. Based on the results of this study, utilizing any SUA (except UB) as advertised by the manufacturer, may lead to bond strength less than the standard of care (silane and a resin cement).

This laboratory study provides insight on manufacturer claims and comparison to typical practice, but there are noted limitations. Small differences in positioning of the shearing force could result in changes in stress distribution (25), and although care was taken to maintain standardization across specimens, some variation would be expected. Universal adhesive systems are marketed for all direct and indirect dental restorative applications. However, limited studies have been published evaluating marketing claims. This is especially true of newer silane-containing universal adhesives and highlights the need for additional high-quality, long-term studies.

## Conclusions

With the application of silane, all the universal bonding agents (except UB) resulted in a greater bond strength of the resin cement to lithium disilicate. Universal Bond had the greatest bond strength when utilized according to manufacturer’s instructions (without silane). All the SUAs without the use of silane, resulted in lower bond strength (except UB), compared to the typical clinical practice of the use of silane alone.

## Figures and Tables

**Table 1:** Instructions for use and components of materials.

Material Name	Manufacturer	Utilized IFUs	Components
Ceramic Etch	Ivoclar	Ceramic etch was applied to the lithium disilicate specimens for 20 seconds and rinsed.	5% hydrofluoric acid
Bis-Silane	Bisco	The Bis-Silane was mixed by dispensing one drop from each of the two bottles (Parts A & B) into a mixing well and stirred. 1-2 coats (thin coats) of Bis-Silane were brushed on to the surface of the etched lithium disilicate specimens. After 30 seconds the specimens were dried with an air syringe.	Part A: 3-(Trimethoxysilyl) propyl-2-Methyl-2Propenoic Acid; ethanol. Part B: ethanol
NX3	Kerr	Dispensed NX3 dual-cure cement into tubing. Seated tubing onto lithium disilicate specimen, allowing cement to flow from all sides. Tack cured (1-2 seconds) to facilitate cleanup. Removed excess cement. Light cured all surfaces for 20 seconds each.	Base: barium aluminoborosilicate glass, ytterbium fluoride, ethoxylated bisphenol-A dimethacrylate, urethane dimethacrylate, triethylene glycol dimethacrylate, hydroxymethyl methacrylate, fumed silica, bisphenol A-glycidyl methacrylate, ethyldimethylaminobenzoate Catalyst: barium aluminoborosilicate glass, ytterbium fluoride, triethylene glycol dimethacrylate, ethoxylated bisphenol-A dimethacrylate, urethane dimethacrylate, fumed silica, bisphenol-A glycidyl methacrylate, hydroxyethyl methacrylate, peppermint oil
Scotchbond Universal (SBU)	3M ESPE	Placed one drop each of Scotchbond Universal and Scotchbond Universal DCA in a mixing well and mixed for 5 sec. Immediately after mixing, used the disposable applicator to apply the adhesive to the surface of the lithium disilicate specimen and allowed it to react for 20 secs. Did not light cure.	10-methacryloyloxydecyl dihydrogen phosphate, dimethacrylate resins, hydroxyethyl methacrylate, methacrylate modified polyalkenoic acid copolymer, filler, ethanol, water, initiators, silane
Scotchbond Universal Plus (SBU+)	3M ESPE	Used the disposable applicator to apply the adhesive to the lithium disilicate specimens to be bonded and rubbed it in for 20 secs. Subsequently directed a gentle stream of air over the liquid for 5 secs until a shiny film appeared that no longer moved in the stream of air. If the adhesive film was not shiny, the adhesive was re-applied and subjected to the air flow. The adhesive was not light cured on the lithium disilicate specimens.	Hydroxyethyl methacrylate, 10-methacryloyloxydecyl dihydrogen phosphate, bisphenol A-glycidyl methacrylate, ethanol, photoinitiator, fillers, water
Clearfil Universal Bond Quick (CU)	Kuraray Noritake	Dispensed one drop each of BOND and Clearfil DC Activator and mixed them. Applied the mixture of BOND and Clearfil DC Activator to the lithium disilicate specimens, then dried by blowing mild air until the mixture did not move (5 sec+). Light cured (10 seconds).	Bisphenol A-glycidyl methacrylate, hydroxyethyl methacrylate, ethanol, 10-methacryloyloxydecyl dihydrogen phosphate, hydrophilic aliphatic dimethacrylate, colloidal silica, camphorquinone, silane coupling agent, accelerators, initiators, water
Tokuyama Universal Bond (UB)	Tokuyama Dental	Dispensed one drop of each Tokuyama Universal Bond A and B into the same dimple of disposable mixing well and mix. Applied the mixed bond (did not wait) to the lithium disilicate specimen. Applied mild (medium) air to the surface (did not light cure).	A: acetone, phosphoric acid monomer, bisphenol A-glycidyl methacrylate, triethylene glycol dimethacrylate, hydroxyethyl methacrylate B: acetone, isopropanol, water, borate catalyst, peroxide, silane coupling agent

**Table 2:** Mean microshear bond strength and standard deviation of the various groups.

Materials	Microshear Bond Strength MPa (SD)	
	Without Silane	With Silane
Scotchbond Universal (SBU)	1.48 (0.43) Bb	14.00 (4.39) Aa
Scotchbond Universal Plus (SBU+)	1.68 (0.42) Bb	8.27 (2.30) Ba
Clearfil Universal Bond Quick (CU)	1.20 (0.53) Bb	12.55 (4.33) Aa
Tokuyama Universal Bond (UB)	12.88 (5.01) Aa	13.93 (3.89) Aa
NX3 Resin Cement (NX3)	0.63 (0.46) Bb (negative control)	5.19 (3.24) Ba (positive control)

Groups with the same upper-case letter per column and lower-case letter per row are not significantly different (*p*>0.05)

## Data Availability

The views expressed are those of the authors and do not reflect the official views or policy of the Uniformed Services University, Department of Defense, or its Components.
